# Activity and interactions of methane seep microorganisms assessed by parallel transcription and FISH-NanoSIMS analyses

**DOI:** 10.1038/ismej.2015.145

**Published:** 2015-09-22

**Authors:** Anne E Dekas, Stephanie A Connon, Grayson L Chadwick, Elizabeth Trembath-Reichert, Victoria J Orphan

**Affiliations:** 1 Division of Geological and Planetary Sciences, California Institute of Technology, Pasadena, CA, USA

## Abstract

To characterize the activity and interactions of methanotrophic archaea (ANME) and *Deltaproteobacteria* at a methane-seeping mud volcano, we used two complimentary measures of microbial activity: a community-level analysis of the transcription of four genes (16S rRNA, methyl coenzyme M reductase A (*mcrA*), adenosine-5′-phosphosulfate reductase α-subunit (*aprA*), dinitrogenase reductase (*nifH*)), and a single-cell-level analysis of anabolic activity using fluorescence *in situ* hybridization coupled to nanoscale secondary ion mass spectrometry (FISH-NanoSIMS). Transcript analysis revealed that members of the deltaproteobacterial groups *Desulfosarcina/Desulfococcus* (DSS) and *Desulfobulbaceae* (DSB) exhibit increased rRNA expression in incubations with methane, suggestive of ANME-coupled activity. Direct analysis of anabolic activity in DSS cells in consortia with ANME by FISH-NanoSIMS confirmed their dependence on methanotrophy, with no ^15^NH_4_^+^ assimilation detected without methane. In contrast, DSS and DSB cells found physically independent of ANME (i.e., single cells) were anabolically active in incubations both with and without methane. These single cells therefore comprise an active ‘free-living' population, and are not dependent on methane or ANME activity. We investigated the possibility of N_2_ fixation by seep *Deltaproteobacteria* and detected *nifH* transcripts closely related to those of cultured diazotrophic *Deltaproteobacteria*. However, *nifH* expression was methane-dependent. ^15^N_2_ incorporation was not observed in single DSS cells, but was detected in single DSB cells. Interestingly, ^15^N_2_ incorporation in single DSB cells was methane-dependent, raising the possibility that DSB cells acquired reduced ^15^N products from diazotrophic ANME while spatially coupled, and then subsequently dissociated. With this combined data set we address several outstanding questions in methane seep microbial ecosystems and highlight the benefit of measuring microbial activity in the context of spatial associations.

## Introduction

Methane is a potent greenhouse gas, and its consumption by microbes in methane seep sediment reduces its release into the overlying water column (Reeburgh, 2007). The oxidation of methane in seep sediments is mediated primarily by three groups of anaerobic methanotrophic archaea (ANME): ANME-1 (Orphan *et al.*, 2002), ANME-2 (Boetius *et al.*, 2000; Orphan *et al.*, 2001b) and ANME-3 (Niemann *et al.*, 2006; Lösekann *et al.*, 2007). Although they can be detected as single cells or monospecific aggregates (Orphan *et al.*, 2002, Lösekann *et al.*, 2007), ANME, and particularly ANME-2 and ANME-3, are typically found in direct physical association with *Deltaproteobacteria*. ANME-2 associate with three distinct groups of putatively sulfate-reducing *Deltaproteobacteria*: (1) SEEP-SRB1, members of *Desulfosarcina/Desulfococcus* (DSS) within the *Desulfobacteraceae* (Boetius *et al.*, 2000; Orphan *et al.*, 2001a); (2) SEEP-SRB2, a deeply branching deltaproteobacterial group originally described as the Eel-2 group (Orphan *et al.*, 2001a; Kleindienst *et al.*, 2012); and (3) SEEP-DBB, within the *Desulfobulbaceae* (DSB) (Pernthaler *et al.*, 2008; Green-Saxena *et al.*, 2014). ANME-3 have been shown to associate with relatives of SEEP-SRB3 within the DSB (Niemann *et al.*, 2006; Lösekann *et al.*, 2007). Although the chemical interaction between ANME-2/-3 and the associated *Deltaproteobacteria* remains an area of active research (Moran *et al.*, 2008; Milucka *et al.*, 2012), the associated *Deltaproteobacteria* are traditionally thought to mediate sulfate reduction, consuming the reduced products of ANME-2/-3 methane oxidation and driving the thermodynamic favorability of the anaerobic oxidation of methane (Hoehler *et al.*, 1994; Boetius *et al.*, 2000; Orphan *et al.*, 2001b; Hallam *et al.*, 2004; Alperin and Hoehler, 2009).

*Deltaproteobacteria*, including DSS and DSB, are also detected as physically independent (i.e., single) cells within methane seep sediment, comprising 5–20% (>10^8^ cells per cm^3^) of the non-ANME-associated population (Knittel *et al.*, 2003; Schreiber *et al.*, 2010; Kleindienst *et al.*, 2012). Although often referred to as ‘free-living' cells, it is not known if single DSS and DSB are active when physically independent of ANME. In one study, it was suggested that the presence of abundant single DSB cells at an oil field site was because of disruption of the ANME–DSB association, rather than the presence of a truly free-living population (Schreiber *et al.*, 2010). If they are active, how their physiology compares with their ANME-associated counterparts, and particularly whether they are dependent on methane, as ANME-associated *Deltaproteobacteria* appear to be (Nauhaus *et al.*, 2002; Dekas *et al.*, 2009, 2014), remain intriguing questions.

Direct characterization of the activity of single versus ANME-associated *Deltaproteobacteria* is challenging, because standard experiments (e.g., sulfate reduction rates, enzyme activity and phylogenetic or isotope analyses of bulk-extracted biomolecules, including DNA, RNA or lipids) cannot differentiate between phylogenetically similar organisms occupying distinct spatial niches. Most studies investigating single seep *Deltaproteobacteria* have focused on their abundance, distribution and phylogenetic identity, without assessing their activity or ecological function (Knittel *et al.*, 2003; Lösekann *et al.*, 2007; Schreiber *et al.*, 2010; Kleindienst *et al.*, 2012). There is therefore a great deal of uncertainty related to what fraction of the single-cell assemblage is active, what their metabolic capabilities entail and if and how they interact with the ANME-*Deltaproteobacteria* consortia.

Recently, nitrogen fixation, the biological conversion of N_2_ to NH_3_, has been observed in methane seep sediment from the Eel River Basin (ERB) and Mound 12 Costa Rica (Dekas *et al.*, 2009, 2014). Although ANME-2 archaea were identified as the primary diazotrophs at Mound 12, a wide diversity of dinitrogenase reductase (*nifH*) gene sequences have been described from Mound 12 as well as other deep-sea methane seeps, raising the possibility that multiple members of the community are able to fix nitrogen (Dang *et al.*, 2009; Dekas *et al.*, 2009, 2014; Miyazaki *et al.*, 2009). Putatively sulfate-reducing *Deltaproteobacteria*, including DSS and DSB, are candidates for additional seep N_2_ fixation, both because some seep-recovered *nifH* sequences show high similarity to those of cultured diazotrophic sulfate-reducing *Deltaproteobacteria*, and beacause N_2_ fixation mediated by sulfate-reducing bacteria has been observed in shallow marine sediments (Bertics *et al.*, 2010, 2012; Fulweiler *et al.*, 2013). Therefore, if single seep *Deltaproteobacteria* are indeed active, they may be a source of bioavailable nitrogen to the seep ecosystem.

Here, we sought to determine if single DSS and DSB cells in methane seep sediment (1) are active, (2) are dependent on methane and/or ANME activity and (3) if they fix nitrogen. To this end, we investigated the activity of bacteria and archaea in sediment collected at Mound 12 Costa Rica in microcosm experiments amended with methane or argon, and either ^15^NH_4_^+^ or ^15^N_2_. We investigated the microbial community composition, activity and response to methane with an analysis of DNA and RNA (rRNA and mRNA). We then measured anabolic activity and/or diazotrophic ability in single DSB, single DSS, ANME-2-associated DSS, ANME-1 and ANME-2 in the presence and absence of methane with fluorescence *in situ* hybridization coupled to secondary ion mass spectrometry (FISH-NanoSIMS). With this combined data set, we were also able to address several additional outstanding questions in seep microbial ecosystems, including if ANME-2 are anabolically active in the absence of methane, whether ANME-1 and ANME-2 display differences in anabolic activity and if there is a phylogenetic diversity of active diazotrophs in Costa Rican seep sediment.

## Materials and methods

### Sample collection

Seafloor sediment push cores investigated in this study were collected using the manned submersible *Alvin* and R/V *Atlantis* in October 2006 (cruise number AT15-11) within methane seep sites in the ERB Southern Ridge (~40°47.192′N, 124°35.706′W; 520 m water depth; 5 °C *in situ* water temperature) and in January 2010 (cruise number AT15-59) at Mound 12, Costa Rica (~8°55.8′N, 84°18.7′W; 988 m water depth; 5 °C *in situ* water temperature). Sediment cores were immediately stored at 4 °C and extruded from push core liners in 3 cm increments on-board within 2 h after recovery of the submersible. Sediment samples were either stored in Mylar bags flushed with argon (Ar) at 4 °C (ERB) or immediately combined with Ar-sparged filtered seawater and aliquoted into anaerobic serum bottles (CR). Push cores used in this study are listed in Supplementary Table 1.

### ^15^N-labeling microcosm incubations

As described in Dekas *et al.* (2009, 2014), sediments were homogenized with Ar-sparged artificial marine media (ERB) or with filtered bottom water collected near core sampling (CR). Sediment slurries were aliquoted into 140 ml (ERB) or 35 ml (CR) serum bottles with butyl stoppers, crimped and amended with methane (CH_4_) or argon (Ar) (to an overpressure of 2 atm), as well as ^15^N_2_ (5.2%, ERB, or 2%, CR, of headspace) or ^15^NH_4_^+^ (2 mm, ERB, or 1 mm, CR, final concentration), and then stored at 4 °C in the dark. ^15^N_2_ gas was supplied by Cambridge Isotopes (Tewksbury, MA, USA; NLM-363, lot no. I1-10077 or I1-10798). Subsamples of sediment slurry were collected via needle and syringe at at 0, 28, 84 and 168 days (ERB), and at 4, 63, 139 and 275 days (CR). Subsamples for FISH were fixed with 2% paraformaldehyde overnight at 4 °C, washed with phosphate-buffered saline (137 mm NaCl, 2.7 mm KCl, 10 mm Na_2_HPO_4_·7H_2_O, 2 mm KH_2_PO_4_) and ethanol, and stored at −20 °C in 100% ethanol. Subsamples for nucleic acid extraction were immediately flash frozen in liquid nitrogen in 2 ml cryovials and stored at −80 °C. Incubation details are summarized in Supplementary Table 1.

A total of 46 incubations were conducted with CR sediment from six push cores. Bulk rates of nitrogen fixation (^15^N_2_ incorporation), anabolic activity (^15^NH_4_^+^ incorporation) and sulfide production were measured over time, and previously reported in Dekas *et al.* (2014). Incubations with the highest rates of nitrogen fixation (CR8 and CR15, both amended with ^15^N_2_ and CH_4_), and incubations from the same sample as CR15 but amended differently (CR17, amended with ^15^N_2_ and Ar, and CR18, amended with NH_4_^+^ and Ar), were selected for further analysis in the current study. CR45 (amended with ^15^N_2_ and Ar) was also analyzed here, to confirm NanoSIMS results observed in CR17. All analyses reported here were conducted on CR incubations, except the FISH-NanoSIMS analysis of ANME-1 cells, which were conducted on ERB incubations, because of low numbers of ANME-1 cells in the CR sediment.

The time points investigated were selected based on trends in ^15^N incorporation and sulfide production and over time: 139-day subsamples (~5 months, Costa Rica incubations) and 168-day subsamples (6 months, ERB incubations). Given the long doubling time of ANME-*Deltaproteobacteria* consortia (estimated 3–7 months; Girguis *et al.*, 2005; Nauhaus *et al.*, 2007; Krueger *et al.*, 2008; Orphan *et al.*, 2009), long incubations times are necessary to observe the synthesis of new biomass via ^15^N incorporation, particularly when N_2_ serves as nitrogen source (Dekas *et al.*, 2009). Non-target, DAPI (4,6-diamidino-2-phenylindole)-stained cells were analyzed to ensure that excessive recycling of substrates did not occur.

### DNA and RNA extractions and reverse transcription reactions

DNA and RNA were extracted simultaneously from 1 ml of frozen sediment slurry using the RNA Powersoil Total RNA Isolation Kit (MOBIO Laboratories, Carlsbad, CA, USA; cat. no. 12866-25) and the RNA Powersoil DNA Elution Accessory Kit (MOBIO Laboratories; cat. no. 12867-25). The extraction was performed according to the manufacturer's instructions, with the following modification: after the addition of solution SR2, the mixture was divided into four 2-ml screw top tubes and cells mechanically lysed using a Bio 101 FastPrep FP120 bead beater (Thermo Electron Corporation, Milford, MA, USA) for 45 s at a speed of 5.5 three times. The RNA extracts were treated with the Ambion TURBO DNA-free Kit (ThermoFisher Scientific, Waltham, MA, USA; cat. no. AM1907), and cleaned using the Qiagen RNeasy Kit (Hilden, Germany; cat. no. 74104), following the RNA Clean-up Protocol provided by the manufacturer. Reverse transcription of RNA to cDNA was completed using Superscript III First Strand Synthesis Supermix (ThermoFisher Scientific; cat. no. 18080-400).

### DNA and cDNA clone libraries

Traditional Sanger sequencing of clone libraries was performed to obtain near full-length 16S rRNA sequences (in contrast to the shorter reads provided by higher throughput alternatives) to enable superior phylogenetic analysis and comparison with regions used in FISH oligonucleotide probe design. Fourteen libraries were generated from RNA and DNA extracted simultaneously from incubations of AD4587 PC6 sediment of the 3–6 cm horizon at the 20-week time point: CR15 (^15^N_2_ with CH_4_ headspace) and CR17 (^15^N_2_ with Ar headspace). Twenty-five microliters of PCR reactions containing 1 μl each of 10 μm forward and reverse primer, 1 μl template (5–16 ng DNA or cDNA), 2.5 μl of 10 × ExTaq PCR buffer (Takara, Clontech Laboratories, Inc., Mountain View, CA, USA), 0.3 μl of 5 U μl^−1^ ExTaq (Takara), 0.5 μl of 10 mm dNTPs (New England Biolabs, Ipswich, MA, USA), 0.5 μl of 10 μg μl^−1^ bovine serum albumin and 18.2 μl water were performed. The following primer sets were used: nifHf_10aa and nifHr_132aa (Mehta *et al.*, 2003) to target dinitrogenase reductase (*nifH*), mcrA_F and mcrA_R modified from (Luton *et al.*, 2002) to target methyl coenzyme M reductase A (*mcrA*), AprA-1-FW and AprA-5-RV (Meyer and Kuever, 2007) to target adenosine-5′-phosphosulfate reductase α-subunit (*aprA*) and 27 F and 1492 R modified from Lane (1991) to target the 16S rRNA gene. Primer sequences are listed in Supplementary Table 2, and PCR conditions in Supplementary Table 3. No amplicon was visible in any of the RNA-only reactions (no-RT reaction) when 4 μl were run on an agarose gel. PCR products were plate purified (Millipore Multiscreen filter plates; ref. no. MSNU03010), ligated with the Invitrogen TOPO TA Cloning Kit (ThermoFisher Scientific; cat. no. K457501) and transformed using Top Ten chemically competent *Escherichia coli* cells. Picked colonies were grown overnight in Luria–Bertani broth and amplified using M13 primers for 30 cycles. The M13 products were visualized to confirm the correct size insert, plate purified and sent for unidirectional sequencing using T3 primers at Laragen Sequencing (Culver City, CA, USA). For the 16S rRNA library, clones that were identified as *Deltaproteobacteria* after unidirectional sequencing were sent for reverse sequencing with T7 primers. All unique, full-length sequences were deposited into GenBank with the following accession numbers: KR813881–KR814285 (16S rRNA), KR020406–KR020496 (*nifH*), KR812737–KR813018 (*aprA*) and KR812576–KR812736 (*mcrA*). Sequences derived from CR15 begin 'CH4–' and sequences derived from CR17 begin 'Ar–'.

### Phylogenetic analysis and identification

All sequences were trimmed, examined for quality and stitched (16S rRNA deltaproteobacterial sequences only) using Sequencher software (Gene Codes Corporation, Ann Arbor, MI, USA). Two 16S rRNA gene trees were inferred by maximum likelihood using PhyML package (Guindon *et al.*, 2010) and the HKY evolutionary model in the software program ARB version 5.5 (Ludwig *et al.*, 2004). The bacterial positional variability filter was provided within the SSURef-111-SILVA-NR database (Quast *et al.*, 2013). The reliability of the trees was estimated by bootstrapping in Geneious version 7.0.4 (Kearse *et al.*, 2012) using PhyML maximum likelihood, the HKY model and 100 replicates. The 16S rRNA identification of all clones was also checked using the SSURef-119-SILVA-NR database. The *aprA* sequences were translated in ARB and aligned with MUSCLE (MUltiple Sequence Comparison by Log-Expectation). The *aprA* phylogeny was computed using MrBayes (Ronquist *et al.*, 2012). Convergence was determined by an average standard deviation of split frequencies <0.01. The *nifH* and *mcrA* sequences were translated in Geneious and aligned using ClustalW. The *nifH* and *mcrA* trees were generated by maximum likelihood (PhyML) using the LG substitution model (Le and Gascuel, 2008), with branch support estimated using the approximate likelihood ratio test (Anisimova and Gascuel, 2006).

### FISH-NanoSIMS

FISH-NanoSIMS was performed on paraformaldehyde-fixed ANME-*Deltaproteobacteria* consortia and/or single cells from CR8, CR17, CR18 and CR45 (139-day time point), and ERB1C and ERB5A (168-day time point) using previously described protocols (Dekas and Orphan, 2011). CR8 rather than CR15 was investigated to pair the single-cell FISH-NanoSIMS analyses performed here with the ANME-2 consortia FISH-NanoSIMS analyses performed previously (reported in Dekas *et al.*, 2014). CR8 and CR15 are analogous incubations: both contained sediment collected under microbial mats from Mound 12, both sediment inoculum horizons were proposed to be the methane–sulfate transition zone based on ANME abundance, their sediment inoculum showed similar diversity in *nifH* sequences and both showed methane-dependent sulfide production and nitrogen fixation (Dekas *et al.,* 2014).

Sediment was centrifuged in a 1:1 phosphate-buffered saline:Percoll gradient (Sigma-Aldrich, St Louis, MO, USA; P4937), followed by filtration on a 0.2-μm polyvinylidene fluoride filter (Durapore Membrane Filter; EMD Millipore, Hayward, CA, USA). Damp filters were flipped onto slides, depositing cells on the slide surface (custom-cut glass squares coated with indium tin oxide or glass rounds). Catalyzed reporter deposition fluorescence *in situ* hybridization (CARD-FISH) was conducted following the protocols in Pernthaler *et al.* (2002) and Pernthaler and Pernthaler (2007) using the following horseradish peroxidase-labeled probes: DSS_658 (Boetius *et al.*, 2000), seepDBB_653 (Green-Saxena *et al.*, 2014) and ANME-1_350 (Boetius *et al.*, 2000). Probe sequences and formamide concentrations are listed in Supplementary Table 2. Tyramides conjugated with Alexa Fluor 488 or Alexa Fluor 546 were used in the CARD-FISH amplification reactions. Hybridizations with DSS_658 and seepDBB_653 were conducted sequentially on the same samples. Cells were counterstained with DAPI. Consortia containing both cells positively hybridized with the DSS_658 probe and unhybridized, DAPI-stained cells were identified as ANME-DSS consortia for NanoSIMS analysis. To support this identification, mono-label FISH experiments (as described in Orphan *et al.*, 2002) were conducted on sediment from the same incubations using probes DSS_658 and EelMS_932 (targeting ANME-2) (Boetius *et al.*, 2000). These experiments demonstrated that >90% of cell clusters containing DSS cells were indeed ANME-2-DSS aggregates (Supplementary Figure 1B). Cells of interest were imaged and mapped using 60 × (PlanApo; Olympus, Shinjuku, Tokyo), 40 × (UPlanFLN; Olympus) and 10 × (Plan-Neofluar; Zeiss, Jena, Germany) objectives on a Delta Vision RT microscope and Softworx software (Applied Precision, Issaquah, WA, USA).

A CAMECA NanoSIMS 50 l housed at Caltech (Pasadena, CA, USA), operated with a mass resolving power of ~5000, was used to analyze specific cells identified with FISH. Cells deposited on glass were gold coated before NanoSIMS analysis. A Cs^+^ primary ion beam (2–8 pA) with a nominal spot size of 100–200 nm was used to rastor over cells of interest. Seven masses were collected: ^12^C^−^, ^13^C^−^, ^14^N^12^C^−^, ^14^N^13^C^−^, ^28^Si^−^, ^31^P^−^ and ^32^S^−^ using electron multipliers. Raster images of 9–100 μm^2^ were collected at 256 × 256 or 512 × 512 pixels resolution, for 0.5–5 h. Clostridia spores with known isotopic composition (previously analyzed by isotope-ratio mass spectrometry) were used as standards. Images were processed using L'Image software (developed by L Nittler, Carnegie Institution of Washington, Washington, DC, USA).

## Results and Discussion

### Combining DNA and RNA Investigations with FISH-NanoSIMS

Investigating the activity of microbes within natural samples is a focus of environmental microbiology, and a key to understanding the role of microbes in biogeochemical cycles. Here, we investigated the occurrence (DNA) and expression (RNA) of four key genes in methane seep sediment from Mound 12 Costa Rica incubated with either CH_4_ or Ar to assess the potential activity of seep microorganisms. We analyzed 16S bacterial rRNA, to target the bacterial community, *aprA*, to target sulfur-cycling microbes, *mcrA*, to target methanotrophs and methanogens and *nifH*, to target organisms capable of N_2_ fixation (diazotrophs).

Although RNA provides a more accurate assessment of microbial activity than DNA, the detection of rRNA and/or mRNA is still an imperfect proxy (see discussion in Blazewicz *et al.*, 2013). Ribosomes can be detected in dormant cells (e.g., Sukenik *et al.*, 2012), and posttranscriptional and posttranslational regulation can lead to decoupling of mRNA expression, protein synthesis and enzyme activity (e.g., Kessler and Leigh, 1999; Liang *et al.*, 1991; Waldbauer *et al.,* 2012; Zhang *et al.*, 1993). Additionally, although RNA in deceased cells is generally thought to degrade quickly, the lifetime of transcripts in the environment—particularly in cold anoxic sediments—is poorly understood, and there is some evidence for survival of RNA post- mortem (Fordyce *et al.*, 2013). It is therefore beneficial to combine community-level RNA analysis with direct measurements of cellular activity when possible.

Consequently, we follow our RNA analysis with direct measurements of microbial anabolic activity using FISH-NanoSIMS. We measure uptake of ^15^N_2_ and ^15^NH_4_^+^ to directly detect diazotrophic activity (N_2_ fixation) and overall anabolic activity, respectively (Krueger *et al.*, 2008; Orphan *et al.*, 2009). In addition to providing a more definitive measure of microbial activity, FISH-NanoSIMS observes activity in the context of spatial associations, allowing the differentiation of activity between phylogenetically similar microbes occupying different spatial niches (e.g., *Deltaproteobacteria* in association with ANME versus physically independent). Despite the benefits of FISH-NanoSIMS analysis, single-cell isotope analysis remains a time-consuming and expensive procedure, precluding the analysis of more than a small subset of cells within a community. The two methods are therefore complimentary: we use the DNA and RNA analysis to obtain a broad community perspective of the organisms present and potentially active under different experimental conditions, and targeted FISH-NanoSIMS analyses to both validate trends seen in our RNA analysis and test specific hypotheses generated by the RNA analysis.

### Methanotroph community composition and activity

**Figure 1:**
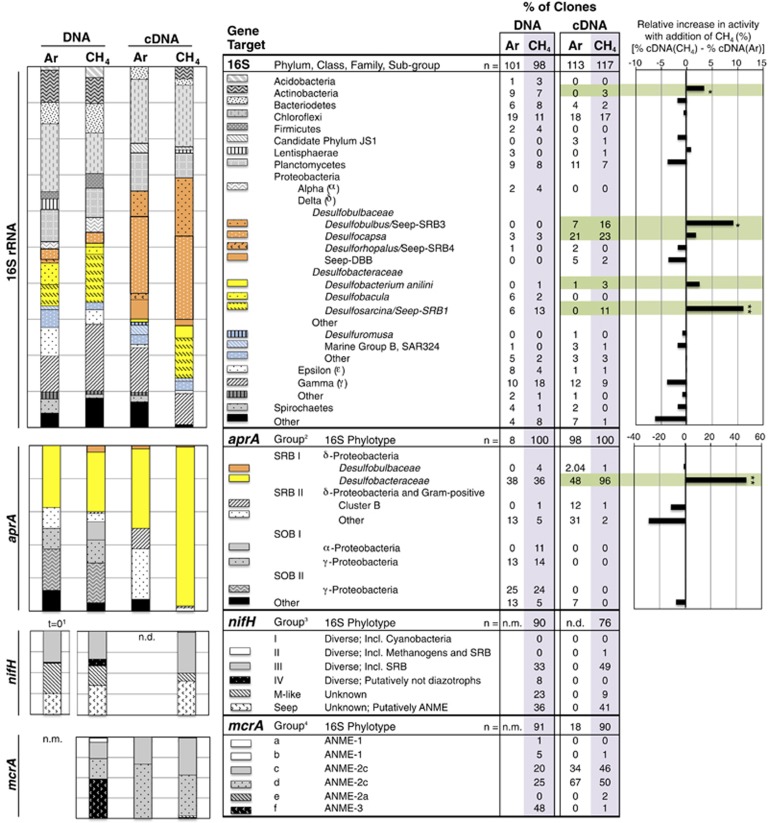
Relative abundance of genes and transcripts detected in 14 clone libraries generated from DNA and cDNA derived from Mound 12 sediment incubated with CH_4_ or Ar. In (**a** and **b**), *Deltaproteobacteria* are in color, with DSB-affiliated sequences in orange and *Desulfobacteraceae*-affiliated sequences in yellow. In (**c**), green bars highlight phylotypes demonstrating higher relative transcription with CH_4_. Asterisks indicate significant increases as determined by a one-tailed *Z*-test of proportions: *95% confidence; **99% confidence; *n*, number of clones sequenced; nm, not measured; nd, none detected. ^1^Not-incubated, *t*=0 sediment (data previously appeared in Dekas *et al.*, 2009); ^2^SRB, sulfate-reducing bacteria; SOB, sulfide-oxidizing bacteria, as defined by Meyer and Kuever (2007); ^3^grouping as defined by Raymond *et al.* (2004); ^4^grouping as defined by Hallam *et al.* (2003).

We detected all three major groups of ANME at Mound 12 (Figures 1a and b). *mcrA* groups a–e have been paired with the 16S rRNA identities of the ANME previously: *mcrA* groups a and b belong to ANME-1, c and d to ANME-2c, e to ANME-2a and f to ANME-3 (Hallam *et al.*, 2003; Lösekann *et al.*, 2007; Meyerdierks *et al.*, 2010; Wang *et al.*, 2014). In the methane incubation, ANME-1-affiliated *mcrA* sequences were recovered from the DNA-based survey (6% of the library), with a single ANME-1-affiliated clone detected in the cDNA library (1% of the library) (Figures 1a and b). ANME-2- and ANME-3-affiliated *mcrA* sequences together dominated the DNA library (46% and 49%, respectively), but transcripts affiliated with ANME-3 were not detected (Figures 1a and b).

*mcrA* transcripts were also detected in the Ar incubation (Figures 1a and b). Notably, PCR amplification of *mcrA* in cDNA from the Ar incubation was significantly less than that from the CH_4_ incubation, determined by visualization of the PCR product via gel electrophoresis. From this library, 18 *mcrA* sequences were obtained, all belonging to the ANME-2c-affiliated group c/d, and highly similar to *mcrA* transcripts recovered from the methane treatment (Figures 1a and b and Supplementary Figure 2). Three primary possibilities may explain the detection of these *mcrA* transcripts in the absence of methane: ANME methanotrophy fueled by *in situ* methane production, methanogenesis by ANME or low turnover of *mcrA* transcripts. Methanogenesis by ANME has been proposed in other sediments based on thermodynamic calculations (Alperin and Hoehler, 2009), as well as environmental observations including the detection of ANME-1 16S rRNA and *mcrA* transcripts within methanogenic zones of sediment cores (Lloyd *et al.*, 2011), direct natural abundance δ^13^C measurements of ANME-1 and ANME-2 cells (House *et al.*, 2009) and contemporaneous detection of methane production and oxidation in ANME-dominated sediments (Orcutt *et al.*, 2005, 2008; House *et al.*, 2009). However, as described above, detection of transcripts does not definitively indicate activity. We therefore employed FISH-NanoSIMS to determine if the detection of ANME-2-affiliated *mcrA* transcripts in the Ar incubation coincided with anabolic activity.

### ANME-DSS consortia: anabolically active in the absence of CH_4_?

**Figure 2:**
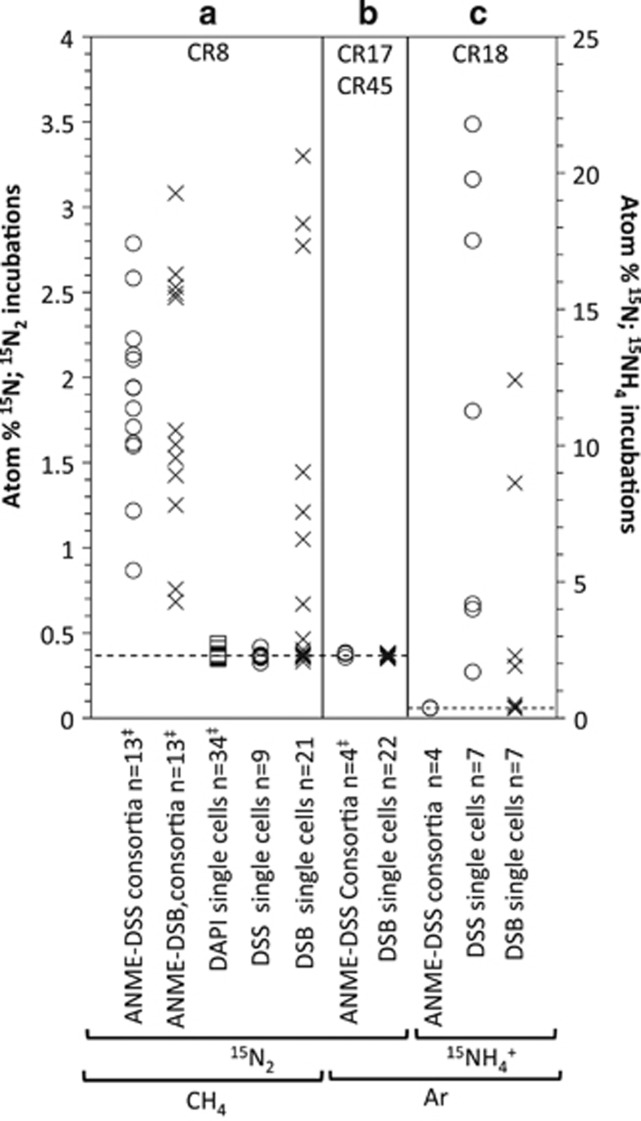
NanoSIMS analysis of ^15^N assimilation from ^15^N_2_ (**a** and **b**, left axis) or ^15^NH_4_^+^ (**c**, right axis) in ANME-DSS consortia, ANME–DSB consortia, unidentified DAPI-stained single cells, DSS single cells and DSB single cells, recovered from Costa Rica Mound 12 sediment after incubation with the indicated amendments. ^‡^Data previously published in Dekas *et al.* (2014), redisplayed here for comparison. The dashed lines indicate natural abundance atom % ^15^N.

**Figure 3:**
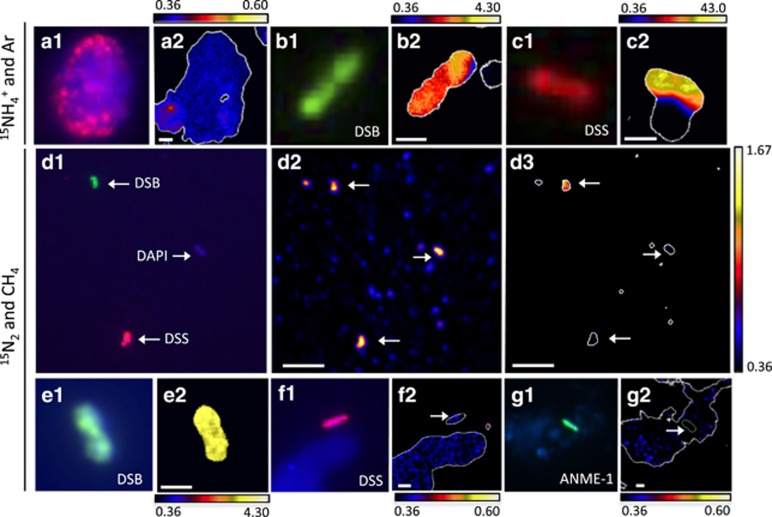
Paired CARD-FISH and NanoSIMS images of methane seep microorganisms incubated with ^15^NH_4_^+^ and Ar (**a**–**c**) or ^15^N_2_ and CH_4_ (**d**–**g**)**. a1, b1, c1, d1, e1, f1, g1:** CARD-FISH images show DSB (probe seepDBB_653), DSS (probe DSS_658) and ANME-1 (probe ANME-1_350), counterstained with DAPI (blue) as indicated. (**a1**) An ANME-2-DSS aggregate with probe DSS_658 in red. **a2, b2, c2, d3, e2, f2, g2:** NanoSIMS images of the same cells show their isotopic (atom % ^15^N) composition. (**d2**) A ^12^C**^−^** ion image of the same cells in (**d1** and **d3**), with ^12^C**^−^** counts ranging from 0 to 900 per pixel. The minimum value for all atom % ^15^N color bars is natural abundance; the maximum varies by image. The isotope images show data only for pixels that exceed a threshold for total ^12^C**^−^** counts (10–30% of the maximum ^12^C**^−^** counts in the image); these areas are enclosed by white outlines and may include more area compared with the cell of interest because of the presence of non-cellular carbon-containing particles and/or extracellular polymeric substance (EPS). In (**g2**), the arrow and green outline indicate the location of the cell, which was drawn by hand, and determined using the ^12^C^15^N^**−**^ and ^32^S^−^ ion images (not shown). Scale bars in (**a2**, **d2** and **d3**) are 5 μm; all others are 1 μm.

To determine if ANME-2-DSS consortia were anabolically active without methane, we measured uptake of ^15^NH_4_^+^ by ANME-2-DSS consortia in the Ar incubation via FISH-NanoSIMS. FISH using oligonucleotide probes EelMS_932 and DSS_658 revealed the occurrence of intact, ribosome-containing ANME-2-DSS consortia in the absence of methane for 9 months, without statistically significant changes in abundance or morphology (CR17 and CR18; Supplementary Figure 1). Previous FISH-NanoSIMS analyses showed no ^15^N incorporation from ^15^N_2_ by ANME-2-DSS aggregates in the absence of methane in incubation CR17 (Dekas *et al.*, 2014), but left open the possibility of non-diazotrophic anabolic activity. Here, we demonstrated that general anabolic activity (^15^NH_4_^+^ uptake) by ANME-2-DSS consortia was also below detection after 14 months of incubation without methane (*n*=4 consortia, including ~550 cells, calculated assuming average cell volume of 1 μm^3^, CR18); (Figures 2c and 3a).

The lack of ^15^NH_4_^+^ uptake demonstrates that although they persist without methane, and are detected by FISH, the ANME-2 and associated DSS cells investigated were anabolically dormant without methane. Anabolic dormancy in this subset of the population suggests that ANME-2-mediated methanogenesis, or methanotrophy fueled by *in situ* methane production, coupled to growth, is not widespread in this community. These results also suggest that ANME-2-associated DSS are typically dependent on ANME activity (and/or directly on methane), and do not fully disassociate or recover the ability to grow independently of ANME/methane even after months in the absence of active methanotrophy. However, because of the targeted nature of FISH-NanoSIMS, even with the analysis of ~550 cells, we cannot eliminate the possibility of ^15^NH_4_ incorporation in a subset of the ANME-2- and/or ANME-associated DSS populations not included in our analysis.

### Sulfur-cycling bacterial diversity and activity

**Figure 4:**
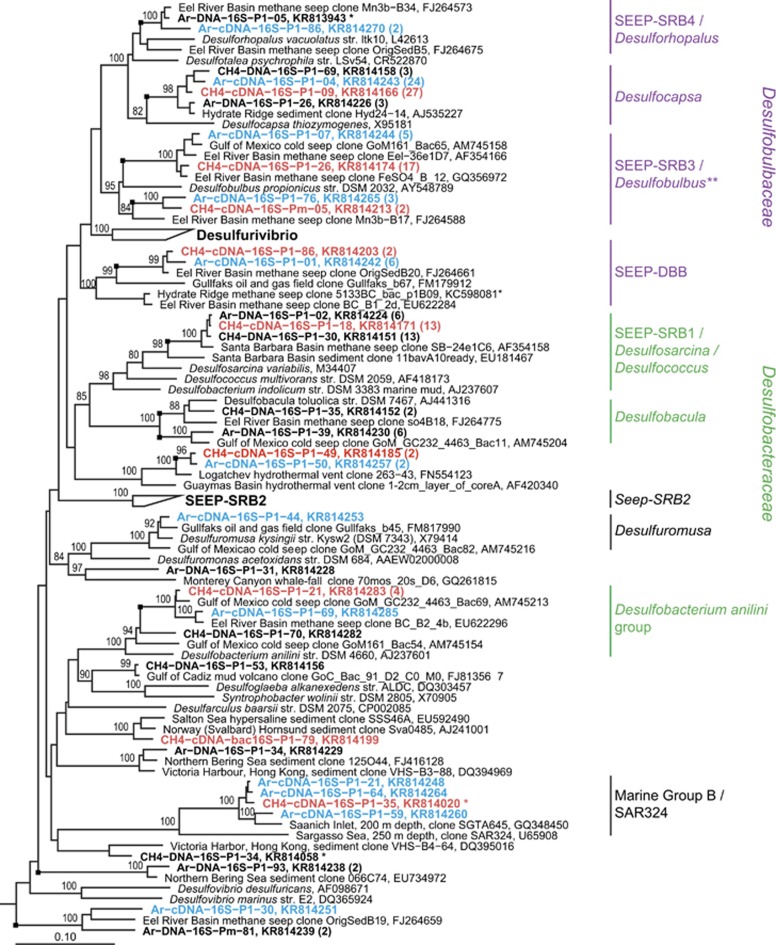
16S rRNA gene tree inferred with maximum-likelihood, HKY evolutionary model and 100 bootstraps. Sequences from this study are in bold. cDNA clones are larger and in color, with clones incubated with CH_4_ in red (incubation CR15), and Ar in blue (incubation CR17). For brevity, only a representative subset of the clones from this study is included in the tree. The total number of clones from each library that fall within the group of the clone shown (defined by the last well-supported branch, and indicated by the black square) is included within parentheses. Purple and green phylogenetic labels indicate DSB and *Desulfobacteraceae*, respectively. Bootstrap support of 70 or greater is shown. The scale bar indicates the average number of nucleotide substitutions per site. One thousand three hundred and seven nucleotides were used to infer the tree. *A shorter sequence (866–987 bp) that was inserted into the tree by parsimony. **A more detailed version of this portion of the tree can be found in Supplementary Figure 4. The tree was rooted with Aquificaceae species AB026268, GU233444, AP011112, AJ005640 and M83548. NCBI accession numbers are shown.

The bacterial 16S rRNA genes recovered from Mound 12 revealed a bacterial assemblage typical of methane seep habitats, including a large fraction of bacteria likely involved in sulfur cycling (Figures 1a and b). We detected three of the four uncultured, seep-specific putatively sulfate-reducing clades: SEEP-SRB1, SEEP-SRB3 and SEEP-SRB4, all of which expressed rRNA (Figure 4). Interestingly although transcripts belonging to the DSB clades SEEP-SRB3 and Seep-DBB were detected in the cDNA (together comprising up to 18% of the cDNA libraries), no SEEP-SRB3 or Seep-DBB genes were detected in the DNA clone libraries (*n*=199 clones) (Figures 1a and b). Their presence and ecological contribution may therefore be overlooked by investigations of DNA alone, using conventional cloning and sequencing methods.

The *aprA* gene sequences recovered were split nearly evenly between those of sulfate-reducing bacteria (*aprA* groups SRB I and II) and sulfide-oxidizing bacteria (*aprA* groups SOB I and II), but the transcripts were dominated by SRB sequences (Figures 1a and b and Supplementary Figure 3). Sulfate reduction is therefore likely more prevalent than sulfide oxidation in these incubations, consistent with the net production of sulfide observed over time (Dekas *et al.*, 2014). Few DSB *aprA* sequences were detected in DNA or cDNA, which is surprising given the abundance of DSB 16S rRNA sequences in the cDNA. Although this could suggest that the *aprA* sequences of the uncultured DSB groups detected do not cluster with the *aprA* sequences of cultured DSB, the disparity may also be because of mismatches in the *aprA* primer set to the aprA sequences of seep DSB. The *aprA* reverse primer contained two or more mismatches to the *aprA* genes within a DSB-linked metagenomic bin from a Hydrate Ridge methane seep (Connor Skennerton and Victoria Orphan, unpublished data). Therefore, the lack of DSB *aprA* sequences cannot be commented on.

### Differences in bacterial expression with methane: ANME-coupled activity?

Because of the important role *Deltaproteobacteria* likely play in the anaerobic oxidation of methane, there is great interest in identifying *Deltaproteobacteria* in metabolic partnerships with ANME. Previous studies have used FISH to visualize bacteria physically associated with ANME (e.g., Boetius *et al.*, 2000; Orphan *et al.*, 2001b; Niemann *et al.*, 2006; Lösekann *et al.*, 2007; Pernthaler *et al.*, 2008; Schreiber *et al.*, 2010; Holler *et al.*, 2011; Kleindienst *et al.*, 2012; Vigneron *et al.*, 2014). In the current study, we utilized a different approach to detect bacterial lineages with ANME-coupled activity, by observing differential 16S rRNA gene expression with and without methane. Bacterial lineages whose metabolisms are positively linked with ANME activity were expected to display higher relative levels of rRNA expression in the presence of methane (i.e., when ANME are active) than without. Unlike FISH, this approach does not require that metabolic partners are physically associated to be detected.

The two bacterial groups with the largest increase in 16S rRNA expression with CH_4_ were SEEP-SRB1 and SEEP-SRB3 (Figure 1c and Supplementary Figure 4). Indeed, SEEP-SRB1 transcripts were only detected with methane, suggesting that Seep-SRB1 are dependent on methane and/or ANME activity. This is consistent with the FISH-NanoSIMS observation that ANME-associated DSS were anabolically dormant without methane (Figure 2), as well as numerous previous reports showing SEEP-SRB1 in association with ANME-2 (Boetius *et al.*, 2000; Orphan *et al.*, 2001a; Pernthaler *et al.*, 2008; Schreiber *et al.*, 2010; Dekas *et al.*, 2014).

Seep-SRB3 have not been observed in direct association with ANME. Members of a sister group to Seep-SRB3 associate with ANME-3 (Lösekann *et al.*, 2007), but the Seep-SRB3 clones demonstrating methane-enhanced transcription here are distinct from the ANME-3 partner (Supplementary Figure 4). Additionally, *mcrA* transcripts affiliated with ANME-3 were not detected in these incubations (Figures 1a and b). The observation of enhanced rRNA expression in the presence of methane, although currently uncorroborated by additional data sets, suggest that members of Seep-SRB3 may benefit from methane and/or ANME-2 activity and warrants further investigation.

### Are single cells of DSS and DSB active, comprising a truly ‘free-living' population?

To differentiate activity between ANME-associated and single *Deltaproteobacteria*, we analyzed ^15^NH_4_^+^ uptake in individual cells with NanoSIMS. We used CARD-FISH with the oligonucleotide probe DSS_658 to target members of DSS (which includes the SEEP-SRB1 group; Knittel *et al.*, 2003; Schreiber *et al.*, 2010), and probe seepDBB_653 to target a broad group within the DSB, including members of SEEP-DBB and SEEP-SRB3/*Desulfobulbus* (Supplementary Table 4). We detect the uptake of ^15^NH_4_^+^ in individual DSS and DSB cells in the absence of CH_4_ (DSS: *n*=7 cells, 7/7 ^15^N-enriched; DSB: *n*=7 cells, 4/7 ^15^N-enriched; CR18). This indicates that both single-cell populations targeted by our FISH probes contain members that are anabolically active and not dependent on either CH_4_ or ANME activity (Figures 2c and 3b, c).

Additionally, compared with the lack of ^15^NH_4_^+^ incorporation in ANME-associated DSS in the same incubation (Figures 2c and 3a), the ^15^NH_4_^+^ incorporation measured in all single DSS cells suggests potentially fundamental physiological and likely phylogenetic differences between ANME-associated and physically independent DSS cells. In support of this, the *aprA* transcripts related to *Desulfobacteraceae* and *Desulfobulbaceae* recovered from the CH_4_ and Ar treatments were largely associated with different clades (Supplementary Figure 3).

### Are diverse diazotrophs present and active in Mound 12 sediment?

**Figure 5:**
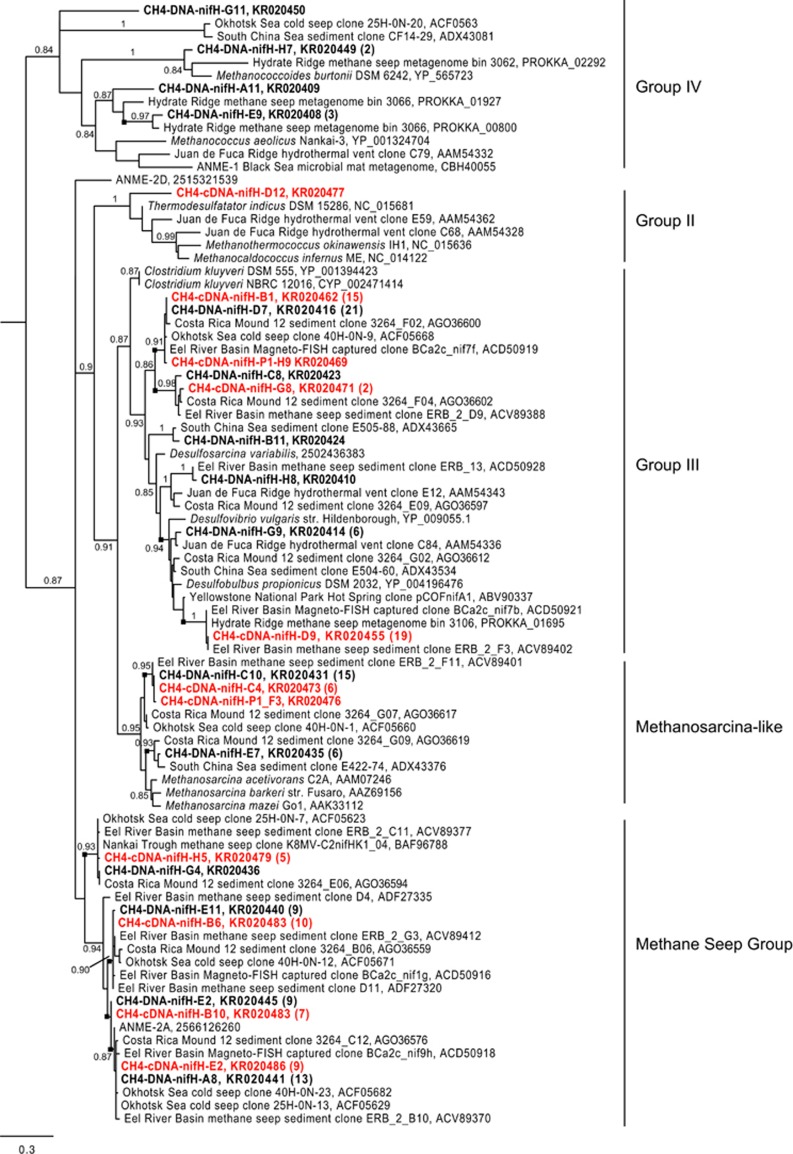
Translated *nifH* gene tree inferred with Maximum Likelihood using PhyML 3.0. Sequences from this study are bold. DNA clones are black and cDNA clones are red. For brevity, only a representative subset of the clones from this study is included in the tree. The total number of clones from each library that fall within the group of the clone shown (defined by the last well-supported branch, and indicated by the black square) is included in parentheses. SH-like aLRT (approximate Likelihood Ratio Test) branch supports above 0.70 for major branches are shown. The scale bar indicates the average number of amino acid substitutions per site. The tree was rooted with chlorophyllide reductase gene sequences YP_004716603, YP_680532, YP_001534851, and YP_508121. NCBI accession numbers are shown when possible; the IMG Gene IDs are listed for ANME-2D, ANME-2 A, and *Desulfosarcina variabilis*. nifH groups as described by Raymond *et al.*, 2004 are indicated.

A diversity of *nifH* sequences was detected in DNA extracted from the CH_4_ incubation (CR15), consistent with *nifH* diversity observed in hotspots of productivity in the deep sea, including methane seeps, mud volcanoes and hydrothermal vents (Mehta *et al.*, 2003; Dang *et al.*, 2009; Dekas *et al.*, 2009; Miyazaki *et al.*, 2009) (Figures 1a, b and 5). A *nifH* DNA clone library was previously generated from the same sediment sample immediately upon collection (not incubated) (Dekas *et al.*, 2014), and the *nifH* diversity and proportions recovered in that library and the library reported here (after 4 months of incubation with CH_4_) are highly similar (Figure 1a). The similarity suggests that the experimental incubation contains a diazotrophic assemblage representative of the *in situ* population.

We detected *nifH* transcripts in the CH_4_ incubation, but not in the Ar incubation, consistent with previous work demonstrating methane-dependent nitrogen fixation at Mound 12 (Dekas *et al.*, 2014) (Figure 1). This suggests that the diazotrophs are either dependent on methane (e.g., methanotrophs) or dependent on the products of the ANME (e.g., ANME-associated *Deltaproteobacteria*). ANME-2 fix nitrogen in methane seep sediment (Dekas *et al.*, 2009), but other seep diazotrophs have not yet been identified. The methane seep *nifH* clade (‘methane seep group 2' in Miyazaki *et al.*, 2009) has been putatively assigned to the ANME-2 archaea (Dekas *et al.*, 2009; Miyazaki *et al.*, 2009), and our analysis supports this assignment with the placement of a *nifH* sequence recovered from an ANME-2a genome within this clade (Wang *et al.*, 2014) (Figure 5). Interestingly, only 41% of the clones recovered in this study fall within the ANME-2-affiliated methane seep clade, whereas 49% fall within group III (49%) (Figure 5). Group III contains *nifH* sequences from a range of anaerobic microbes, including methanogens and *Deltaproteobacterial* sulfate-reducing bacteria. The transcription of these sequences suggests multiple methane-dependent diazotrophs within the sediments, potentially including ANME phylotypes in addition to ANME-2, or SRB with ANME-coupled activity.

Nitrogen fixation by ANME-associated *Deltaproteobacteria* is one possibility. DSS and DSB associated with ANME demonstrate ^15^N enrichment in the presence of ^15^N_2_ and methane (Dekas *et al.*, 2009, 2014). Although this may be because of N sharing with the diazotrophic ANME-2, additional nitrogen fixation by the associated *Deltaproteobacteria*—although at lower rates—is also possible. Consistent with this, the *nif* genes recovered from nearly purified ANME-2c consortia in previous work were affiliated with both *Methanosarcina*-like and deltaproteobacterial *nif* sequences (Pernthaler *et al.*, 2008). Why closely associated partners would both fix nitrogen is unclear, and to our knowledge, unprecedented. It raises the possibility that nitrogen fixation in these organisms is not because of N limitation, which could be overcome by an N_2_-fixing symbiont, but rather for other benefits only realized by the diazotroph itself. Further analyses, including metatranscriptomics/metaproteomics of individual ANME-*Deltaproteobacteria* consortia and/or immunolabeling of nitrogenase would be necessary to differentiate between the possibilities of N sharing between the diazotrophic ANME and associated *Deltaproteobacteria*, versus independent nitrogen fixation in both.

### Are ANME-1, single DSS and single DSB capable of N_2_ fixation?

To investigate whether the diversity of *nifH* transcripts could be because of a diversity of diazotrophs in the single-cell population, we investigated ^15^N_2_ assimilation in single ANME-1, DSS and DSB cells using FISH-NanoSIMS. ANME-1 contain nif homologs, and although they are inferred to be non-functional in nitrogen fixation based on their phylogenetic placement within *nifH* group IV (Meyerdierks *et al.*, 2010), we directly tested their ability to fix nitrogen in this study. Sediment from the ERB previously shown to host diazotrophic ANME-2 was chosen for this experiment rather than Mound 12 sediment because the incubated ERB sediment contained more ANME-1 cells (incubation ERB1C) (Dekas *et al.*, 2009). NanoSIMS analysis revealed that single cells of ANME-1 did not fix nitrogen after a 6-month incubation with methane and ^15^N_2_ (*n*=10; Figure 3g and Supplementary Figure 5A). However, when we investigated the general anabolic activity of ANME-1 cells in parallel incubations with ^15^NH_4_^+^ and CH_4_, no ^15^N uptake was observed, suggesting these archaea were not active in the microcosm incubation (*n*=5; Supplementary Figure 5B). Conclusions regarding the ability of the ANME-1 to fix nitrogen therefore cannot be drawn. Consistent with previous studies (Nauhaus *et al.*, 2005), the lack of NH_4_^+^ assimilation by ANME-1 in the same incubations where ANME-2 readily assimilated NH_4_^+^ (Dekas *et al.*, 2009) suggests differences in the ecological physiology and optimal growth conditions for members of the ANME-1 and ANME-2.

Single sulfate-reducing bacteria are other likely candidates for N_2_ fixation in methane seep sediment, given their diazotrophic activity in shallow marine sediments (Bertics *et al.*, 2010, 2012). Indeed, the majority of the *nifH* transcripts recovered in this study fall within a phylogenetic clade that includes *nifH* sequences affiliated with deltaproteobacterial sulfate-reducing bacteria (Figure 5). Single DSS and DSB cells were therefore targeted with FISH-NanoSIMS to determine whether or not they fix N_2_. However, after incubation with CH_4_ and ^15^N_2_, single DSS cells were not enriched in ^15^N (*n*=9; CR8). The lack of ^15^N_2_ incorporation observed for the single DSS does not eliminate the possibility of N_2_ fixation, but it suggests that if occurring, it is rare.

Interestingly, 33% of the single DSB cells analyzed did show ^15^N enrichment (*n*=21; CR8) (Figures 2a and 3d–f). Additional DAPI-stained microorganisms from the same incubation showed no ^15^N enrichment, indicating that recycling of ^15^N-enriched substrates was not significant over the course of the 5-month incubation (*n*=34; CR8) (Figures 2a and 3d). Without additional information, the ^15^N enrichment observed specifically in single DSB cells after incubation with ^15^N_2_ and methane would suggest diazotrophy. However, when FISH-NanoSIMS analyses were performed on single DSB cells in paired ^15^N_2_ incubations without CH_4_, surprisingly no ^15^N enrichment was observed (*n*=12, CR17; *n*=10, CR45) (Figure 2b). N_2_ fixation dependent on CH_4_ is unexpected for DSB, especially given that ^15^NH_4_^+^ uptake by the DSB was not CH_4_ dependent (Figure 2c).

Two scenarios could explain the pattern of ^15^N observed in the DSB single cells. The first is that free-living members of the DSB—although not the whole population—are both methane-dependent and diazotrophic. Although methane dependence in free-living SRB is unprecedented, some single DSB did not assimilate ^15^NH_4_^+^ without methane, leaving open the possibility of a methane-dependent sub-population. The second, and perhaps more parsimonious, is that single DSB cells are ^15^N-enriched because of a previous association with active ANME. In a previous study, DSB cells in association with ANME-2 were shown to be ^15^N-enriched after incubation with ^15^N_2_ and CH_4_ (Dekas *et al.*, 2014). This could be due either to direct consumption of ^15^N_2_ (diazotrophy) when in association with ANME-2 or passage of fixed ^15^N products from diazotrophic ANME-2. If followed by disassociation from ANME, both scenarios would result in ^15^N-enriched single DSB cells only in the presence of methane, as we observed here. However, in the latter possibility, the DSB are not diazotrophic. Because of this, the combination of observations does not definitively provide evidence for DSB diazotrophy.

The data may, however, suggest an interesting difference between the ANME-2-DSS and ANME-2-DSB associations. The potential ANME-2-DSB dissociation could either be part of the life cycle of the ANME-2-DSB symbiosis or it could be an indication of a fragile association disrupted by the sampling procedure. Either way, this transient and/or fragile association is different from that observed for the DSS, which although always were ^15^N-enriched when associated with ^15^N-enriched ANME (Dekas *et al.*, 2014) were never observed as ^15^N-enriched single cells in diazotrophic conditions (Figure 2a). The stability of the ANME-DSS connection was also observed in the incubation with argon and ^15^NH_4_^+^: although ANME-associated DSS cells were not ^15^N-enriched, all single DSS analyzed in the same incubation were. The ANME-2-DSS association may therefore either be more stable or physically stronger, compared with that of ANME-2-DSB.

## Conclusions

The results yielded by FISH-NanoSIMS and transcript analysis of sediments from Mound 12 Costa Rica were consistent in some but not all cases (summarized in Supplementary Table 5). The inconsistencies likely resulted from the limitations of each method: transcripts do not definitely indicate activity and lack spatial information, and FISH-NanoSIMS measures activity (or lack thereof) in only a subset of the population. However, in combination, we were able to use these techniques to demonstrate that (1) single DSS and DSB cells are active within methane seep sediment, and are not dependent on methane and/or ANME activity, and (2) single DSS and ANME-associated DSS exhibit physiological differences with respect to their response to methane, suggesting phylogenetic differences. Additionally, we made observations suggesting that (1) a diversity of methane-dependent diazotrophs are active in Mound 12 sediment and may include ANME-associated *Deltaproteobacteria*, (2) the ANME-DSS association is stable, and may be more so than the ANME–DSB association, (3) ANME-1 and ANME-2 demonstrate differing growth rates and/or acceptable growth conditions and (4) Seep-SRB1, and more surprisingly, Seep-SRB3 show a positive transcriptional response to methane/ANME activity. Taken together, these insights provide new understanding of the dynamics between ANME and seep *Deltaproteobacteria*, and highlight the complimentary nature of transcript and FISH-NanoSIMS analyses to assess microbial activity.
