# Supplementary material for: Activity and interactions of methane seep microorganisms assessed by parallel transcription and FISH-NanoSIMS analyses
Source: ISME J. 2015 Sep 22;10(3):678–92. doi: 10.1038/ismej.2015.145 (PMC4817681; doi:10.1038/ismej.2015.145)
Supplement: Supplementary Figure 1 [file ismej2015145x1.pdf]

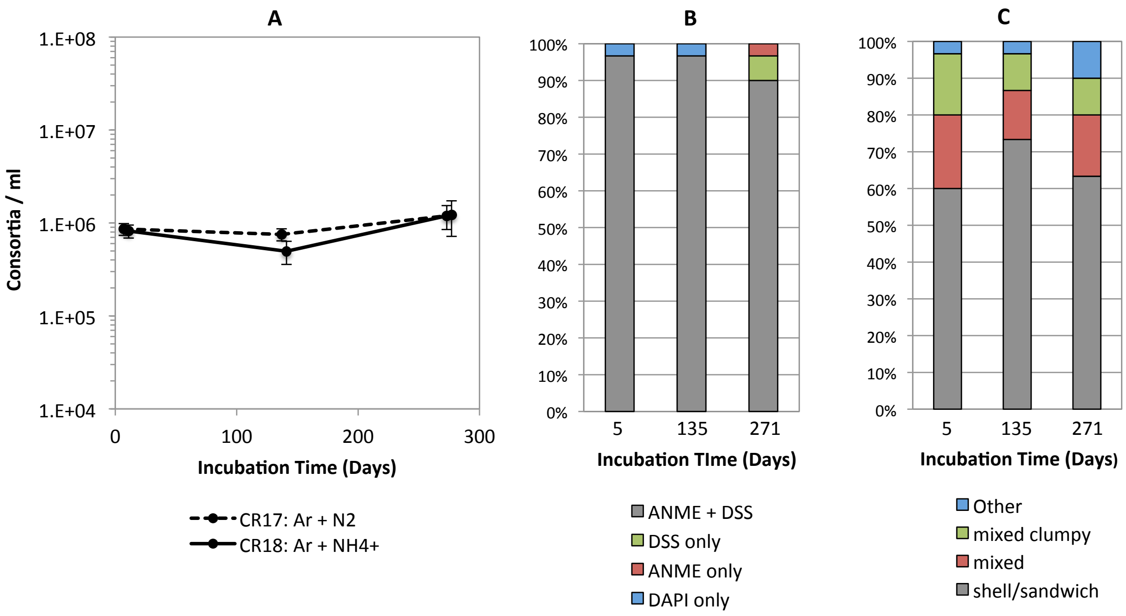

SI Figure 1. Persistence of ANME-DSS consortia in the absence of methane. Change in consortia abundance (A), composition (B), and morphology (C) is shown. Consortia were hybridized with FISH probes EelMS_932 (targeting ANME-2) and DSS_658 (targeting *Desulfocarsina/Desulfococcus*/Seep-SRB1). A: Error bars are one standard deviation calculated from triplicate filters. B and C: Thirty aggregates from incubation CR17 were evaluated for composition and morphology at each time point.
