# Supplementary figures and images for: Activity and interactions of methane seep microorganisms assessed by parallel transcription and FISH-NanoSIMS analyses
Source: ISME J. 2015 Sep 22;10(3):678–92. doi: 10.1038/ismej.2015.145 (PMC4817681; doi:10.1038/ismej.2015.145)

SI Figure 2.

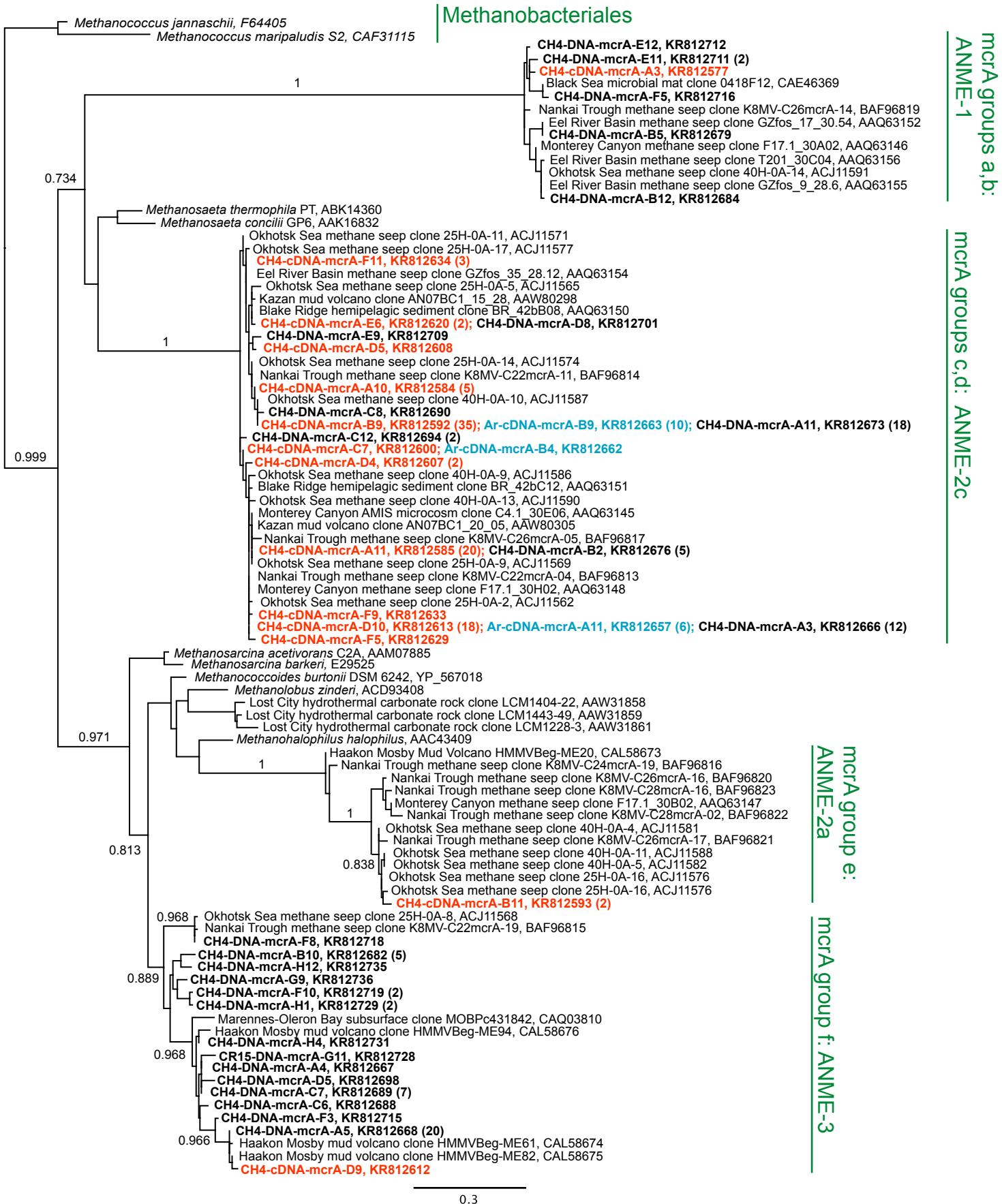

Supplement: Supplementary Figure 2 [file ismej2015145x2.pdf]

## SI Figure 3

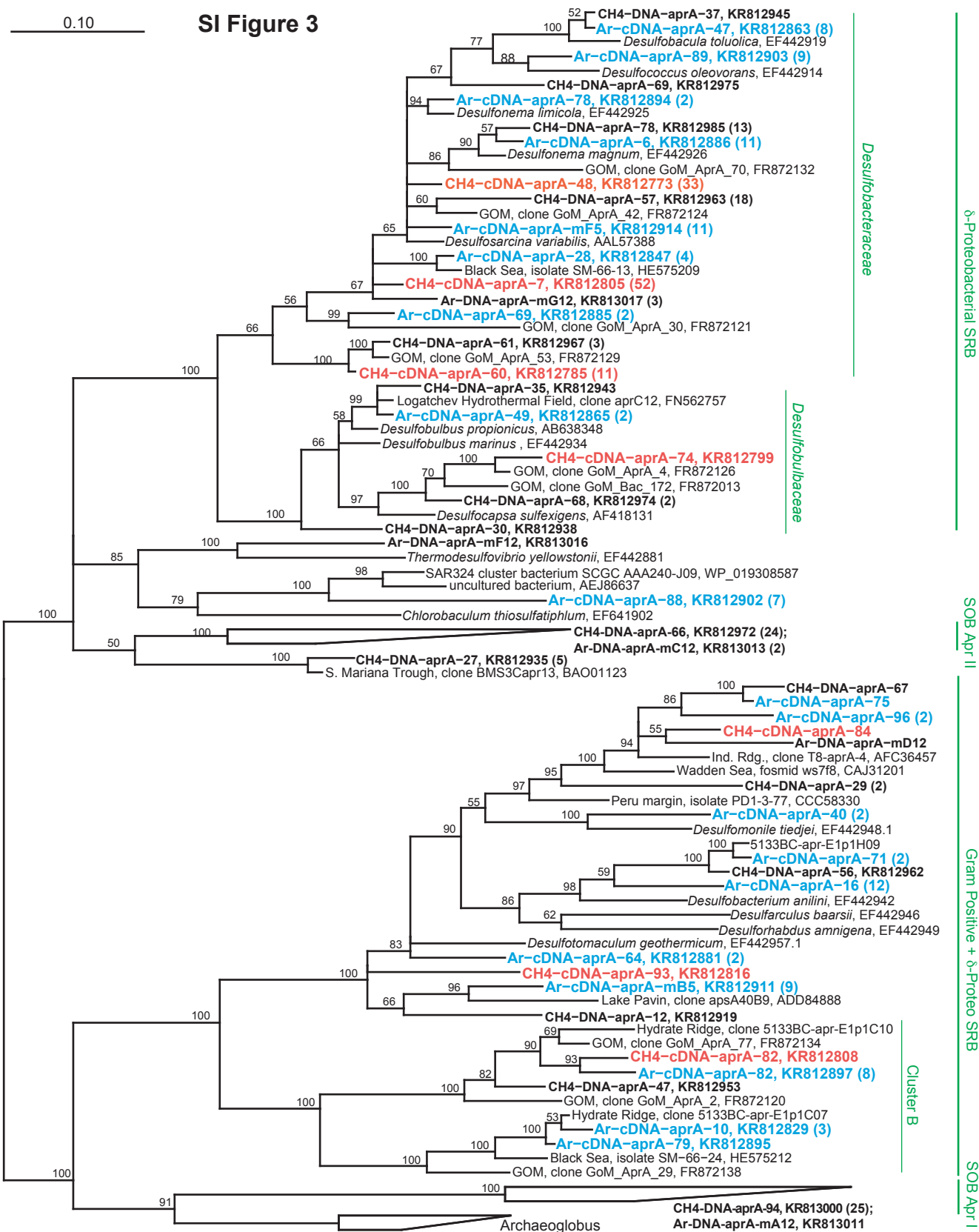

Supplement: Supplementary Figure 3 [file ismej2015145x3.pdf]

SI Figure 4.

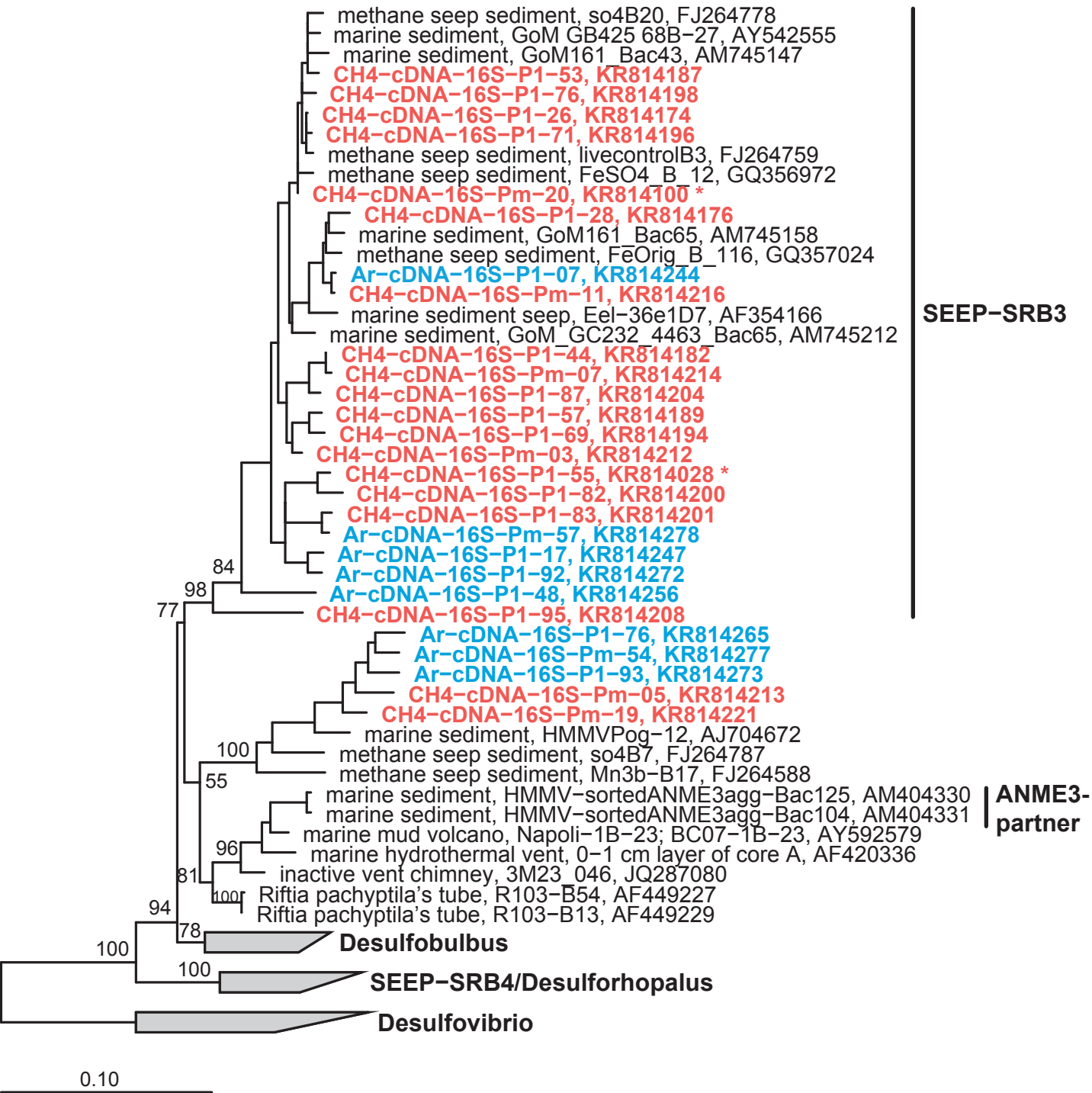

Supplement: Supplementary Figure 4 [file ismej2015145x4.pdf]

SI Figure 5

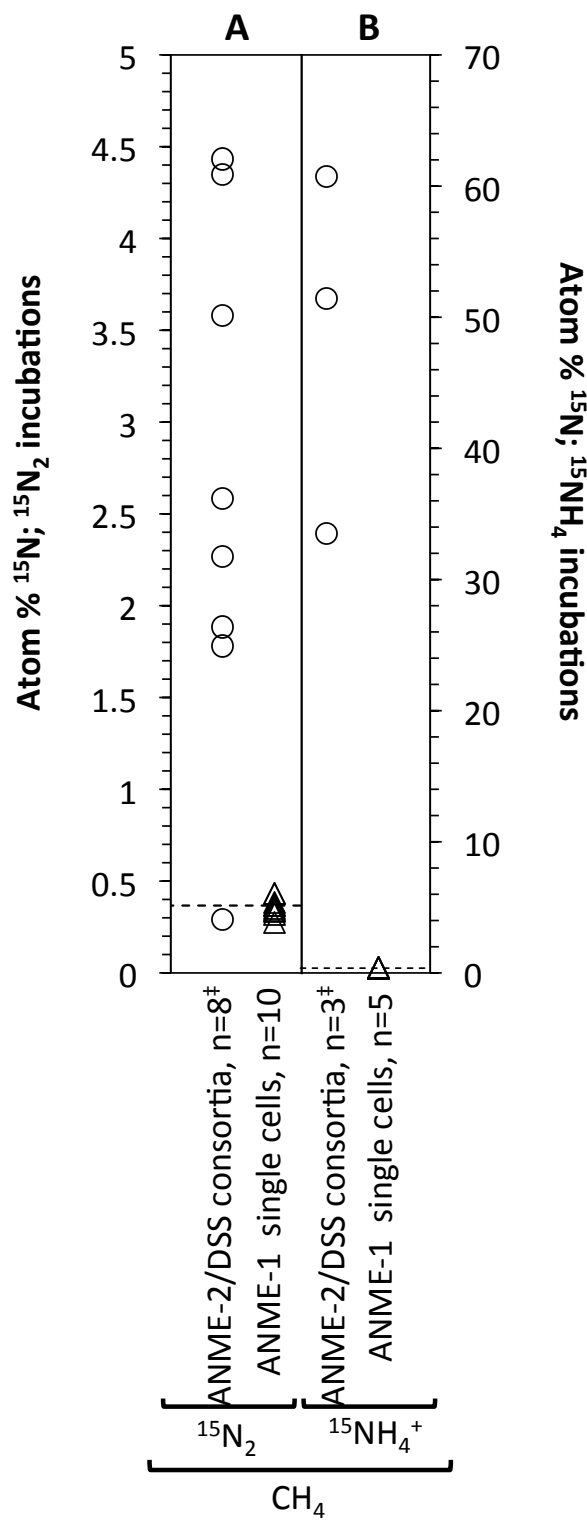

Supplement: Supplementary Figure 5 [file ismej2015145x5.pdf]
