# Supplementary material for: Activity and interactions of methane seep microorganisms assessed by parallel transcription and FISH-NanoSIMS analyses
Source: ISME J. 2015 Sep 22;10(3):678–92. doi: 10.1038/ismej.2015.145 (PMC4817681; doi:10.1038/ismej.2015.145)
Supplement: Supplementary Table 5 [file ismej2015145x10.docx]

| ­Question | DNA | RNA | CARD-FISH | NanoSIMS | Summary |
| --- | --- | --- | --- | --- | --- |
| Are single DSS cells present and active? Independent of CH_4_/ANME activity? | DSS genes are detected | rRNA: DSS transcription is 100% CH_4_-dependent | Single DSS cells are detected | Single DSS cells ­­incorporate ^15^NH_4_^+^ w/ and w/o CH_4_ | Single DSS are present, active, and not directly dependent on CH_4_ or ANME activity. |
|  |  | mRNA: *aprA* transcripts affiliated with *Desulfobacteraceae* are expressed w/o CH_4_ |  |  |  |
| Are single DSB cells present and active? Independent of CH_4_/ANME activity? | DSB genes are not detected | rRNA: DSB transcription is enhanced by CH_4_, but not dependent | Single DSB cells are detected | Single DSB cells incorporate ^15^NH_4_^+^  w/ and w/o CH_4_ | Single DSB are present, active, and not directly dependent on CH_4_ or ANME activity. |
| Do single DSS and DSB cells fix nitrogen? | Diverse *nifH* genes are detected, some are SRB-affiliated. | mRNA: Diverse *nifH* transcripts are detected, and cluster with ANME-2- and SRB-affiliated *nifH* sequences. | N/A | Single DSS did not incorporate ^15^N_2_. | Definitive evidence for diazotrophy by single DSS or single DSB was not observed. The combination of evidence leaves open the possibility for single DSB diazotrophy in a subset of the population that is also CH_4_-dependent , and/or for single DSB that only fix nitrogen when in association with functional ANME, and then dissociate. However, these possibilities are not considered as likely as non-diazotrophic DSB cells acquiring reduced N-products from diazotrophic ANME when in association, and then disassociating. |
|  |  | However, *nifH* transcription is CH_4_-dependent, and evidence reported in this study shows single DSS and DSB are not CH_4_-dependent. |  | Some single DSB are enriched in ^15^N when incubated with ^15^N_2_ and CH_4_, but the enrichment is CH_4_-dependent . Since DSB cells did not show anabolic CH_4_-dependent in general, this suggests ^15^N-sharing from ANME rather than *a priori* diazotrophy. |  |
| Is there a diversity of active diazotrophs in seep sediment? | Diverse *nifH* genes are detected | mRNA: Diverse *nifH* transcripts are detected, and cluster with ANME-2- and SRB-affiliated *nifH* sequences. Transcription is CH_4_-dependent. | N/A | Definitive N_2_ fixation was not observed in ANME-1, single DSS, single DSB, or unidentified DAPI-stained single cells. | ANME-2 continue to be the only diazotrophs specifically identified within seep sediment. However, the transcription of diverse *nifH* sequences suggest that more species are capable, just not yet identified. In particular, the methane dependence of *nifH* transcription suggests the possibility of other ANMEs or ANME-associated SRB. |
| Are ANME-2 anabolically active w/o CH_4_? | N/A | mRNA: ANME-2-affiliated mcrA transcripts detected w/o CH_4_ | ANME-2-DSS consortia persist w/o CH_4_ | ANME-2-DSS consortia do not assimilate NH4^+^ w/o CH_4_ | ANME-2 in association with DSS are generally not anabolically active w/o CH_4_, but the possibility that a minority of the population (undetected by NanoSIMS here) remains active cannot be eliminated. |
| Are ANME-2-associated DSS anabolically active w/o CH_4_? | N/A | rRNA: DSS transcription is 100% CH_4_-dependent | ANME-2-DSS consortia persist w/o CH_4_ | ANME-2-DSS consortia are not anabolically active w/o CH_4_ | ANME-2-associated DSS are generally not anabolically active w/o CH_4_, but the possibility that some remain active cannot be eliminated. |
| Are there differences in activity between ANME-1 and ANME-2? | More ANME-2 affiliated mcrA genes were detected than ANME-1 (CR) | mRNA: ANME-2 have a higher transcript:gene ratio than ANME-1 (CR) |  | ANME-2 assimilate ^15^NH_4_^+^ in ERB sediment but ANME-1 do not | Evidence from both Costa Rica seep sediment (transcripts) and Eel River Basin seep sediment (FISH-NanoSIMS) suggests ANME-2 are more anabolically active than ANME-1 under the conditions employed. |

**SI Table 5.** Major questions addressed by the transcript and NanoSIMS analyses and a comparison of the relevant observations provided by each technique. Observations that are consistent with a positive answer are in green, observations consistent with a negative answer are in red.
