# Supplementary material for: Activity and interactions of methane seep microorganisms assessed by parallel transcription and FISH-NanoSIMS analyses
Source: ISME J. 2015 Sep 22;10(3):678–92. doi: 10.1038/ismej.2015.145 (PMC4817681; doi:10.1038/ismej.2015.145)
Supplement: Supplementary Information [file ismej2015145x11.docx]

**SI Table 1.** Sediment samples and incubation conditions. Color bar on the left identifies incubations from the same site (Costa Rica in blue, Eel River Basin in green) and specific sample (shades of blue ). Red indicates analyses reported in this study.

**SI Table 2.** Primers and probes used in this study.

SI Table 3. PCR conditions used in this study. Assuming complete conversion of RNA to cDNA, concentration of templates (determined by Nanodrop spectrometer analysis of the DNA or RNA) were as follows: CR15 DNA: 16.2 ng/μl; CR15 cDNA: 6.7 ng/μl; CR17 DNA: 12.4 ng/μl, and CR17 cDNA: 5.0 ng/μl.

**SI Table 4.** Number of mismatches between CARD-FISH probes used in this study (DSS_658 and seepDBB_653) and deltaproteobacterial 16S rRNA sequences detected. Green indicates the likely targets of each probe: 0 mismatches for DSS_658, 0 and 1 mismatches for seepDBB_653 (different cut-offs due to the different formamide concentrations in the hybridization reaction for each). Probe DSS658 has been shown to cross-hybridize with probe SEEP2-658, despite 2 differences in the probes (at the second and third positions from the end), suggesting that under some circumstances probe DSS658 may hybridize with sequences containing 2 mismatches (Kleindienst et al 2012). However, no SEEP-SRB2 sequences were detected in this study, and a higher concentration of formamide was used (60% rather than 50%), decreasing the likelihood of unspecific DSS658 binding here. Probe seepDBB_653: CTTTCCCCTCCGATACTC. Probe DSS_658: TCCACTTCCCTCTCCCAT.

**SI Table 5.** Major questions addressed by the transcript and NanoSIMS analyses and a comparison of the relevant observations provided by each technique. Observations that are consistent with a positive answer are in green, observations consistent with a negative answer are in red.

**SI Figure 1.** Persistence of ANME-DSS consortia in the absence of methane. Change in consortia abundance (**A**), composition (**B**), and morphology (**C**) is shown. Consortia were hybridized with FISH probes EelMS_932 (targeting ANME-2) and DSS_658 (targeting *Desulfocarsina/Desulfococcus/Seep-SRB1*). A: Error bars are one standard deviation calculated from triplicate filters. B and C: Thirty aggregates from incubation CR17 were evaluated for composition and morphology at each time point.

**SI Figure 2.** Phylogenetic analysis of *mcrA* sequences. Deduced amino acid sequences were aligned using ClustalW and the phylogeny was computed using Maximum Likelihood in PhyML. Sequences from this study are in bold. cDNA clones are in color, with clones derived from CH_4_ incubations in red, and Ar incubations in blue. Representative sequences are shown; parentheses indicate number of sequences 99.3% similar to the clone shown. SH-like aLRT (approximate Likelihood Ratio Test) branch supports above 0.70 for major branches are shown. mcrA groups as described by Hallam et al 2003 are indicated. NCBI accession numbers are shown.

**SI Figure 3.** Phylogenetic analysis of *aprA* sequences. Deduced amino acid sequences were aligned in Arb with MUSCLE, and the phylogeny was computed using MrBayes. Sequences from this study are in bold. cDNA clones are in color, with clones derived from CH_4_ incubations in red, and Ar incubations in blue. Parentheses include the number of sequences a clone represents. Branch supports as posterior probabilities are shown. aprA groups as described by Meyer and Kuever 2007 are indicated. NCBI accession numbers are shown, except for the following (whose numbers are not included on the tree due to space constraints): CH4-DNA-aprA-67 (KR812973), Ar-cDNA-aprA-75 (KR812891), Ar-cDNA-aprA-96 (KR812908), CH4-cDNA-aprA-84 (KR812810), Ar-DNA-aprA-mD12 (KR813014), CH4-DNA-aprA-29 (KR812937), Ar-cDNAaprA-40 (KR812858), Ar-cDNA-aprA-71 (KR812888), and Ar-cDNA-aprA-16 (KR812835).

SI Figure 4. Phylogenetic analysis of 16S rRNA genes of all clones recovered in this study that affiliate with or near Seep-SRB3 and the previously reported ANME-3 partner (Lösekann et al 2007, Niemann et al 2006). Tree was inferred with Maximum Likelihood, HKY evolutionary model and 100 bootstraps. Sequences from this study are in bold. cDNA clones derived from CH_4_ incubations in red, and Ar incubations in blue. All bootstrap support values are shown on the nodes that separate the clades of interest. Scale bar represents 0.10 substitutions per site and 1271 nucleotides were used to infer the tree. Asterisk indicates shorter sequences (807 and 848 bp) that were inserted by parsimony. NCBI accession numbers are shown.

**SI Figure 5.** NanoSIMS analysis of ^15^N-assimilation from ^15^N_2_ (panel **A**, left axis) or ^15^NH_4_^+^ (panel **B**, right axis) in ANME-2-DSS consortia and ANME-1 single cells recovered from Eel River Basin methane seep sediment after incubation with CH_4_. ^‡^Data previously published in (Dekas et al 2009), re-displayed here for comparison. The dashed lines indicate natural abundance atom % ^15^N.
